# A case of coinfection of a pediatric patient with acute SARS-COV-2 with MIS-C and severe DENV-2 in Mexico: a case report

**DOI:** 10.1186/s12879-021-06765-6

**Published:** 2021-10-18

**Authors:** Perez-Mendez Maria Jose, Zarate-Segura Paola, Davila-Gonzalez Eduardo, Servin-Monroy Monroy Osvaldo Arturo, Bastida-Gonzalez Fernando

**Affiliations:** 1 Laboratorio de Medicina Traslacional, Escuela Superior de Medicina, Instituto Politecnico Nacional, Salvador Díaz Mirón esq. Plan de San Luis S/N, Miguel Hidalgo, Casco de Santo Tomas, 11340 Mexico, CDMX Mexico; 2 Laboratorio de Biología Molecular, Laboratorio Estatal de Salud Pública del Estado de México, Tollocan S/N Colonia Moderna de la Cruz, 50180 Toluca,, Mexico; 3 Departamento de Epidemiología del Instituto de Seguridad Social del Estado de México y Municipios, ISSEMyM, Av. Hidalgo Pte. No. 600, Col. La Merced, 50080 Toluca, Estado de México Mexico

**Keywords:** Case report, Coinfection, COVID-19, DENV-2, Pediatric patient, Severe DENV, MIS-C

## Abstract

**Background:**

COVID-19 cases have been increasing since the epidemic started. One of the major concerns is how clinical symptomatology would behave after coinfection with another virus.

**Case presentation:**

In this case report, a pediatric native patient from Estado de Mexico (EDOMEX), MEX had severe DENV-2 and acute SARS-CoV-2 at the same time. The clinical features were severe thrombocytopenia, secondary septic shock, cerebral edema, pericardial effusion, fluid overload that exhibited bipalpebral edema in all four extremities, hemophagocytic lymphohistiocytosis (HLH), coronary artery ectasia (CAE), multisystemic inflammatory syndrome in children (MIS-C), and probable COVID-19 pneumonia or acute respiratory distress syndrome (ARDS) that triggered patient intubation. The patient presented unusual symptomatology according to the literature. After 15 days of intubation and 15 more days under surveillance, he was released without respiratory sequelae and without treatment after major clinical improvement.

**Conclusion:**

The aim of this manuscript is to present clinical challenges that coinfection may cause in pediatric patients, even though COVID-19 in children does not tend to be as severe as in other sectors of the population.

## Background

In late 2019, many cases of atypical pneumonia were reported in the province of Wuhan, Hubei, China; later, these cases were later linked to a new virus, SARS-CoV-2 [1]. It has now progressed to cause a global pandemic, with 57,882,183 confirmed cases and 1,377,395 deaths reported as of 22 November 2020 [2].

Dengue virus (DENV) is endemic in Mexico, and climatic diversity provides fertile ground for the transmission of DENV, with four serotypes circulating in the territory [3]. At the same time of the COVID-19 epidemic, the dengue season started (June to October). In Mexico, coinfection of SARS-CoV-2 and DENV-2 in pediatric patients has not yet been reported. The aim of this brief report is to present clinical complications, the treatments used, sequelae, and preparation to manage the well-being of children in relation to more incidents of this kind.

## Case presentation

In August 2020, a 7-year-old male patient presented with clinical symptoms of difficulty swallowing, fever of 39 °C, and headache and was diagnosed with bacterial pharyngitis by a primary health care provider. Antibiotics (amoxicillin) and ibuprofen were prescribed. He was subsequently treated at two more private practices as symptoms did not improve, first for gastroenteritis medicated with trimethoprim/sulfamethoxazole and ibuprofen and later with clindamycin, ibuprofen, and nimesulide. Although a slight improvement was seen, the headache worsened and was accompanied by adynamia and asthenia, which were present for at least 5 days. Finally, after 6 days of failed treatments, the patient was admitted to the “Hospital Materno Infantil,” ISSEMYM, EDOMEX, MEX.

During physical examination upon the patient’s admission (illness day 6, admission day 1, D1), petechia was seen in the face, armpits, and lower limbs; he also presented with dehydration and odynophagia. Laboratory tests indicated severe thrombocytopenia (reference range (RR) 150,000–450,000/μL) with a platelet count of 15,000/μL, long prothrombin time (RR 12.2–15.5 s) of 10.7 s, normal hemoglobin (RR 11.5–15.5 g/dL) of 13.9 g/dL, elevated leucocytes (RR 5.0–14.5 × 10^3^/μL) of 16,820/μL, low lymphocyte count (RR 15–61%) of 11.2% (1260/μL), and a normal monocyte count (RR 0–5%) of 3%. Based on clinical suspicion, COVID-19 and DENV RT-qPCR were requested.

On D1, the patient was administered two doses of saline solution at 0.9% due to low cardiac output and was in the 5th percentile for blood pressure. Norepinephrine was also started; however, he did not improve, and eventually presented with secondary septic shock on day 7 of illness (from D2 to D7). During this time, he also had severely stiff limbs and myoclonus, leading to suspicion of encephalitis. A CT scan of the brain showed cerebral edema, which was treated with phenytoin sodium (20 mg/kg loading dose maintained at 7 mg/kg per dose); later, the patient responded to treatment for myoclonus, and he did not present abnormal movements afterwards.

On day 2 of hospital admission, the nasopharyngeal swab result was positive for SARS-CoV-2. Nucleic acid extraction was performed with the chemagic 360 instrument (PerkinElmer, Waltham, MA, USA) using 300 µL of sample in viral transport medium (VTM), amplification was done with SARS-CoV-2 RT-PCR vitro master diagnostica® (Vitro, S.A., Sevilla, Spain), and viral load was quantified in a QX ONE Droplet Digital PCR (ddPCR) System (Bio-Rad, Hercules, CA, USA) which calculated 9.11 copies/µL. The patient was treated with 3rd generation cephalosporin and meningeal doses of ceftriaxone and acyclovir intravenously for 5 days for COVID-19. On the same day, a pediatric cardiology service ran echocardiography, which detected right coronary ectasia and pericardial effusion without hemodynamic compromise; physicians suggested 3 mg/kg acetylsalicylic acid every 24 h, although due to thrombocytopenia, treatment was delayed until D11. He also showed fluid overload, exhibiting bilateral periorbital edema and edema in all four extremities, hepatomegaly, and pericardial effusion and was administered furosemide (D2–16). In addition, on illness day 7, the patient presented with respiratory distress (oxygen saturation 90%), and noninvasive ventilation was initiated due to its clinical evolution. As the inflammatory response continued, multisystemic inflammatory syndrome in children (MIS-C) was suspected due to persistent fever (1 week), headache, cytopenia, thrombocytopenia, anemia, hyperferritinemia, generalized petechia, and elevated d-dimer. He was treated with immunoglobulin gamma (1 g/kg per dose with 2 doses), which is the most effective therapy for MIS-C, from day 7 of illness to day 12 of illness.

Following D4 (day 9 of illness), his condition became unstable due to ventilatory deterioration (respiratory frequency 55 RPM, respiratory acidosis). To prevent worsening, mechanical ventilation was started while he was sedated with opioid analgesia (midazolam 20 mcg/kg/min) until D14 of hospital admission. By D5, clindamycin was added to the COVID-19 treatment.

By D7, the patient had high blood pressure (above the 90th percentile) and was treated with hydralazine (0.2 mg/kg per dose every 6 h) while norepinephrine was stopped, although he presented low cardiac output along with grade III cardiomegaly and was treated with levosimendan (0.1 μg kg/min until D12). Additionally, the patient had electrolyte imbalance (hypernatremia) which was corrected immediately with the Holliday-Segar equation.

On D9 of hospital admission, the patient had hypophosphatemia (RR mild from 2 to 2.5 mg/dL) at 2.3 mg/dL, hyponatremia (RR < 135 mEq/L) at 133 mEq/L, hypomagnesemia (RR < 1.5 mg/dL), and hypokalemia (RR < 3 mEq/L) which were corrected immediately with the Holliday-Segar equation.

On D10, hemophagocytic syndrome was suspected due to the presence of cytopenia, persistent fever, hypertriglyceridemia, and hyperferritinemia.

From D4 to D11, the patient was transfused multiple times due to active bleeding with platelet apheresis or platelet concentrates and was observed to be improving until D11. On D12, clindamycin was still administered as a result of a persistent inflammatory response.

By D12, an increase in D-dimer was detected, and the pediatric hematology service suggested low molecular weight heparin with enoxaparin 1 mg/kg every 24 h. By D14 of hospital admission, the edema had resolved. However, chest X-ray still showed pulmonary congestion, cardiomegaly, and hepatomegaly. At this point, the patient was extubated with supplementary oxygen along with nasal cannulas (1 LPM) and nebulized with ipratropium bromide.

On D15 after hospital admission, the bleeding stopped, but a grade 2 pressure ulcer was noted on the occipital region. A serum sample result was positive for DENV-2. Because of the increase in COVID-19 samples, the state laboratory was at full capacity and samples other than COVID-19 were delayed. Nucleic acid extraction was performed with the chemagic 360 instrument using 300 µL of serum sample, amplification was done with a one-step superscript III and CDC DENV-1–4 Real-Time RT-qPCR Multiplex Assay, and viral load was quantified by a QX ONE Droplet Digital PCR (ddPCR) System (Bio-Rad) and calculated to be 6.36 copies/µL.

On D17 after hospital admission, the patient had pain upon physical examination, and he was also weak and needed support for daily living activities. Petechiae were still present, and hair loss was noted. Some laboratory parameters were still abnormal, including high leucocytes (RR 5.0–14.5 × 10^3^/μL) at 14.84 × 10^3^/μL, high neutrophils at 82%, low lymphocytes (RR 15–61%) at 12%, low erythrocytes at 3.60 × 10^6^/μL, low hemoglobin (RR 11.5–15.5 g/dL) at 11.0 g/dL, and low hematocrit (RR 35–45%) at 30%. By D20 after hospital admission, the patient was moved from the COVID area to the general pediatric ward; at this time, the patient was on supplementary oxygen delivered via a nasal cannula.

Ten days later, the patient had improved clinically, although petechiae were still seen, the pressure ulcer was healing, oxygen saturation was maintained above 90% without supplementary oxygen, and the parameters on the full blood count had normalized to leucocytes at 2,170/μL, neutrophils at 76%, and lymphocytes at 11%.

Finally, on D29, the patient was discharged, and vital signs were in the 50–90th percentile; moreover, respiratory frequency was then under normal percentiles > 90%, and nonsystemic inflammatory response (CRP, ESR, and PCT) and bleeding had stopped. Upon discharge, acetylsalicylic acid (3 mg/kg per dose every 24 h) and prazosin (0.4 mg/kg per dose) 2 mg every 6 h were continued for 2 weeks. After 2 weeks, the patient had fought off the infections and did not have respiratory sequelae, so he was released without treatment and experienced major clinical improvement over 3 months of follow-up.

## Discussion and conclusion

A few cases of coinfection with SARS-CoV-2 and DENV-2 have been reported in different countries for adult patients [4–6], yet this is the first case in a child. As described above, this 7-year-old boy under our care experienced many serious complications during these two infections.

According to previous reports [7–10], the clinical profiles of non-severe dengue disease in children include fever, myalgia, abdominal pain, petechiae, vomiting, retroorbital pain, and abdominal distension. In the case of severe dengue, platelets < 150,000 cells/mm^3^, leukopenia, positive tourniquet, myalgia/arthralgia, petechia, gastrointestinal bleeding, elevation in serum glutamic oxaloacetic transaminase (SGOT), hepatomegaly, fluid leak, and pleural effusion [11] are common symptoms; bilateral effusion, abdominal pain, abnormal prothrombin time (PT), activated partial thromboplastin time (aPTT), and neurological symptoms such as encephalopathy and convulsions are very rare. Some forms of atypical manifestations of severe dengue in children include acute respiratory distress syndrome (ARDS), persistent shock, and diastolic dysfunction. Another secondary clinical consequence of DENV disease is hemophagocytic lymphohistiocytosis (HLH). HLH is a life-threatening condition with severe hyperinflammation from uncontrolled proliferation of active lymphocytes and histiocytes that secrete high amounts of inflammatory cytokines; this condition can be inherited or acquired [12] and has been increasingly reported as a complication of dengue. An analysis of pediatric patients in Puerto Rico showed that dengue patients with HLH between 1 and 13 years old were more likely to be hospitalized; in addition, most cases had a coinfection (hepatitis B virus, *Plasmodium vivax*, *Plasmodium falciparum*, scrub typhus, invasive aspergillosis, herpes simplex virus, respiratory syncytial virus, or Epstein‐Barr virus [13]), longer duration of fever, lymphadenopathy, hepatomegaly, splenomegaly, anemia, and elevated liver transaminases [14]. It was also observed that severe dengue with HLH diagnosis has a higher rate of mortality [15]. In the same way, another relevant yet unusual clinical symptom of DENV is that it can generate cardiomegaly because DENV can infect the heart, myocardial endothelium, and cardiomyocytes [16, 17]. Cardiomegaly was significantly related to elevated blood pressure [18, 19]. Likewise, cardiomegaly has been reported to be observed in patients with COVID-19, with the majority of patients having hypertension and other cardiovascular diseases [20]. Both viruses can generate cardiomegaly mostly in adults with cardiac disease, and infection with both DENV and SARS-CoV-2 can exacerbate cardiovascular complications. In this case report, the patient presented cerebral edema, pericardial effusion, bilateral periorbital edema, edema in all four extremities, hepatomegaly, thrombocytopenia, long prothrombin time, cardiomegaly, and HLH, which are characterized by DENV, although not all symptoms.

In the case of COVID-19, clinical symptoms in pediatric patients are not well documented, although various studies report fever and cough as the main symptoms; others include rhinorrhea, sore throat, headache, fatigue/myalgia, and gastrointestinal symptoms. It was also shown that infants 0–1 years old are more likely to be affected [21]. COVID-19 is characterized by low WBCs, marginally elevated lymphocytes in the early stages, and mild symptoms. In contrast, severe cases of COVID-19 may present with increased serum inflammatory markers, such as D dimer, procalcitonin, creatine kinase, and interleukin 6, as well as progressive lymphopenia [21, 22]. In the early days of the infection, our patient showed mild symptoms, which worsened each day. According to a systematic review [23] in which 7,780 pediatric patients with COVID-19 from 131 studies and 16 countries were evaluated, 19.3% of the children were asymptomatic, and only 3.3% required ICU admission. In contrast, immunosuppressed children or those with chronic cardiac or respiratory illness comprised children with underlying medical conditions (65%). The production of an inflammatory cascade causing multisystemic inflammatory syndrome in children (MIS-C) (11 patients) can create the need for respiratory assistance. The cause of MIS-C is still unclear, but it is disproportionately frequent among African American and Afro-Caribbean populations. The most common symptoms of MIS-C are fever, abdominal pain/diarrhea, and vomiting, while the common laboratory investigation findings are elevated mean neutrophil percentage and low lymphocytes, increased C-reactive protein, ferritin, and procalcitonin, and abnormal d-dimer [24–26].

COVID-19 has also been related neurological symptoms [27]. One of these neurological manifestations is myoclonus. In a systematic review by Chan et al. [28], 51 cases were identified as myoclonus associated with COVID-19, with ages ranging from 26 to 88 years. Myoclonus usually onset within 1 month of COVID-19 symptoms and was multifocal or generalized with a duration from 1 day to 2 months. Treatments involved different medications from anti-epileptic (levetiracetam, clonazepam, valproic acid, and primidone), immunotherapy (methylprednisolone, intravenous immunoglobulin, and plasma exchange), sedative (midazolam, lorazepam, ketamine, and dexmedetomidine), or nonepileptic treatments, with 80% improvement. There has not been a case of a pediatric patient with myoclonus reported, although opsoclonus associated with myoclonus has been reported after COVID-19 in an infant treated with intravenous immunoglobulin and corticosteroids [29]. In this case, the patient presented myoclonus after 1 week of onset of symptoms and was treated with phenytoin sodium. Phenytoin is helpful in only a minority of patients, it has also been related to exacerbated myoclonus seizures, and it only has value in the treatment of cortical myoclonus with motor seizures (palatal myoclonus) [30]. In addition to myoclonus, COVID-19 has been demonstrated to cause cerebral edema by the direct invasion of SARS-CoV-2 to the central nervous system (CNS) [31]. No case with simultaneous edema and myoclonus has been reported. Likewise, cerebral edema, limb stiffness, and limb paralysis are other neurological complications caused by COVID-19. In three different cases, a 66-year-old man, a 52-year-old man, and 61-year-old female with flaccid lower extremity paralysis were associated with acute flaccid myelitis related to COVID-19 [32–34]. In this case report, neither myelitis nor Guillain-Barré syndrome was detected in the pediatric patient, although the latter has been related to COVID-19 and affects myelin in the peripheral nerves, leading to paresis, muscle weakness, and even bilateral ascending paralysis [35]. The limb stiffness and myoclonus in this case may have been generated by COVID-19 or as a consequence of brain edema [36].

A few cases of COVID-19 and dengue have been reported. In a study of 178 adult patients (> 18 years of age) in Brazil, 63% were diagnosed as positive for SARS-CoV-2 after RT-qPCR confirmation; from this percentage, 38.4% of patients were also positive for dengue coinfection by a dengue IgM test. A higher rate of pulmonary impairment and hospitalization were demonstrated in coinfection. The patients also showed lower levels of blood lymphocytes (30.52% vs 26.37%) and monocytes (7.76% vs 6.99%) [37].

Neither COVID-19 nor DENV disease has been shown to cause coronary artery ectasia (CAE). Degradation of the medial arterial layer by activation of serine proteinase in addition to matrix metalloproteinase (MMP) in the arterial smooth muscle cells generates CAE [38]; a study by Shi et al. [39] measured the levels of matrix metalloproteinase 3 in serum, comparing noninfected and infected patients with COVID-19, and found a significant difference in serum MMP3. Patients with COVID-19 have an increase in this enzyme, although a laboratory analysis of MMP3 in this case report was not available. COVID-19-induced elevations of MMP3 causing CAE is a hypothesis worth exploring.

Other relevant points are the high values of white blood cells (WBCs), as DENV disease causes leukopenia according to diverse studies [40, 41]. In the case of COVID-19, leukopenia and lymphopenia have been associated with severe COVID-19 and a negative outcome, mostly in adults [42, 43], although other studies confirmed that high white blood cells (WBCs) in patients with a medical history of myocardial infarction had higher leucocyte counts. Both lymphopenia and high WBC count have been correlated with increased C-reactive protein and mortality [44].

Infection caused by SARS-CoV-2 has been a major challenge for physicians due to its wide range of clinical manifestations. Diverse clinically approved drugs have been tested as potential anti-SARS-CoV-2 candidates [45]. Antiviral drugs such as lopinavir/ritonavir (LPV/RTV) [46], remdesivir (RDV) [47], acyclovir ribavirin, umifenovir, azithromycin, and oseltamivir have been employed to treat SARS-CoV-2, although it is not yet clear whether the use of these antiviral agents is beneficial in improving the outcome of COVID-19 patients [45, 48]. In this case report, the patient was treated with acyclovir. Acyclovir is an agent with antiviral activity against herpes simplex virus (HSV). In a review of 90 articles, 11 articles reported laboratory-confirmed COVID-19 and HSV in 28 patients (7–28 age range) with HSV reactivation. Patients received antiviral therapy against HSV and COVID-19 management, with no mortality reported. The authors concluded that acyclovir could be considered a potential add-on treatment to the COVID-19 treatment regimen, although further clinical trials should be done [49]. On the other hand, bacterial pathogens are an important cause of morbidity and mortality in viral respiratory tract infections. The prevalence, incidence, and characteristics of bacterial infection in patients with SARS-CoV-2 are not well understood and documented. As antibiotics are infective against SARS-CoV-2, they are prescribed in patients with COVID-19 due to the difficulty of ruling out bacterial coinfection or the possibility of bacterial secondary infection during the illness. The broad-spectrum antibiotics used are fluoroquinolones and third-generation cephalosporins in most cases [50]. The pediatric patient in this case report was treated with third-generation cephalosporin and clindamycin to avoid coinfection or secondary infection with pathogenic bacteria.

It is worthwhile to address contraindications of nonsteroidal anti-inflammatory agents (NSAIDs), as these drugs can potentially increase bleeding risk [11]. The patient received high doses after hospitalization. During hospitalization, the patient was prescribed acetylsalicylic after thrombocytopenia was stopped on day 16 of illness, although dengue results weren´t available at that moment and the medication was stopped as soon the positive result was received; the previous doses of acetylsalicylic (after and during hospitalization) might have triggered bleeding. These drugs could have been avoided if rapid tests for DENV had been done on time; unfortunately, hospital and laboratory capacity was limited due to the COVID-19 pandemic with a surplus of patients admitted, so rapid tests were not available.

The patient in this case report did not have all the symptomatology required for MIS-C caused by SARS-CoV-2, and he presented a coinfection with DENV-2, which may have aggravated the severity in both diseases resulting in effects not usually seen in children.

As the COVID-19 vaccine is still in trials and DENV is a national public health concern in Mexico, we may see more cases of coinfection of SARS-CoV-2 and DENV in pediatric patients in the near future; thus, the intention of this paper is to yield insight on behavior, management, and preparation for coinfection. As observed in this case report, not all medication given to this patient was appropriate, thus some symptoms could have been avoided. As clinicians, it is our duty to research the best options for our patients and their wellbeing, especially with a novel virus that is not well characterized in different scenarios. We hope that this case report will help physicians better treat coinfection between DENV and SARS-CoV-2 in pediatric patients.

## Data Availability

The datasets used and/or analyzed during the current study are available from the corresponding author upon reasonable request.

## Abbreviations

aPTT: Activated partial thromboplastin time
ARDS: Acute respiratory distress syndrome
CAE: Coronary artery ectasia
CNS: Central nervous system
COVID-19: Coronavirus disease 2019
CRP: C-reactive protein
DENV: Dengue virus
DENV-2: Dengue virus type 2
EDOMEX: Estado de Mexico
ESR: Erythrocyte sedimentation rate
HLH: Hemophagocytic lymphohistiocytosis
HSV: Herpes simplex virus
ISSEMYM: Instituto de Seguridad Social del Estado de México y Municipios
LPV: Lopinavir
MEX: Mexico
MIS-C: Multisystemic inflammatory syndrome in children
MMP: Matrix metalloproteinase
NSAIDs: Nonsteroidal anti-inflammatory agents
PCT: Procalcitonin
PT: Prothrombin time
RR: Reference range
RT-qPCR: Quantitative reverse transcription polymerase chain reaction
RTV: Ritonavir
SARS-CoV-2: Severe acute respiratory syndrome coronavirus 2
SGOT: Serum glutamic oxaloacetic transaminase
VTM: Viral transport medium
WBC: White blood cells
